# Supplementary material for: Mechanism-based rescue of Munc18-1 dysfunction in varied encephalopathies by chemical chaperones
Source: Nat Commun. 2018 Sep 28;9:3986. doi: 10.1038/s41467-018-06507-4 (PMC6162227; doi:10.1038/s41467-018-06507-4)
Supplement: Supplementary file 1 — Supplementary Information [file 41467_2018_6507_MOESM1_ESM.pdf]

***Supplementary Figures for***

# Mechanism-based rescue of Munc18-1 dysfunction in varied encephalopathies by chemical chaperones

Guiberson et al.

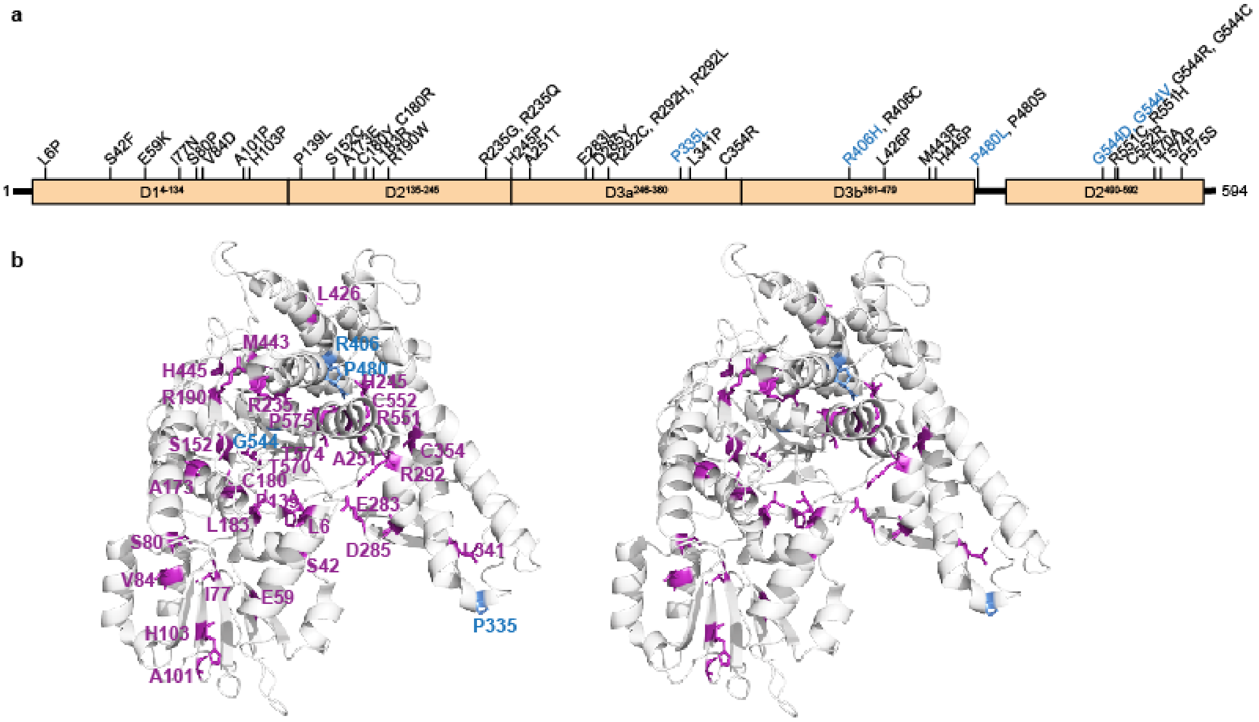


**Supplementary Figure 1. Disease-causing mutations in Munc18-1.** (**a**) Primary sequence of Munc18-1b with indication of its domain structure and positions of disease-linked missense mutations. Highlighted in blue are missense mutations analyzed in this study. (**b**) Localization of disease-causing missense mutations of Munc18-1b in its tertiary structure (PDB code 4JEU[1]), with (left) or without (right) annotation of residues. Highlighted in blue are residues analyzed in this study. Protein structure image was generated using the PDB file and PyMOL (Schrödinger)

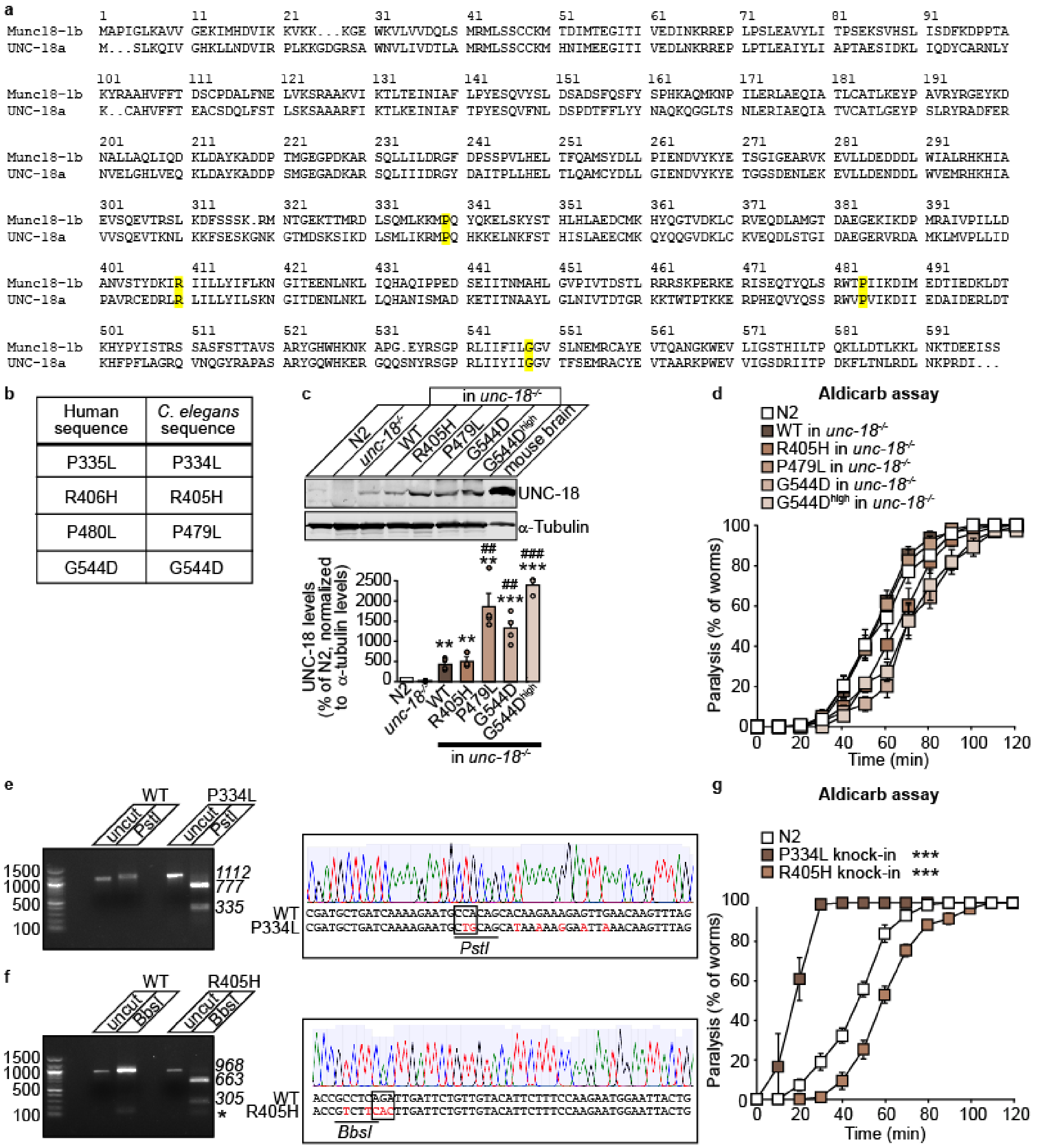


**Supplementary Figure 2. Homologous mutations in human Munc18-1 and *C. elegans* UNC-18 and generation of CRISPR/Cas9-edited knock-in worms.** (**a**) Sequence alignment of human Munc18-1b and *C. elegans* UNC-18a. Highlighted in yellow are the conserved residues analyzed in this study. (**b**) Missense mutations in Munc18-1b and UNC-18a. Indication of analogous missense mutations in the human and worm sequence that were analyzed in this study. (**c**) Expression levels of UNC-18 in *C. elegans*. Total levels of UNC-18 were analyzed by quantitative immunoblotting, normalized to α-tubulin levels. Mouse brain homogenate was separated at the same time, because the Munc18-1 antibody bound non-specifically to other proteins in worm

homogenates. Data are means ± SEM (**,## $p < 0.01$, ***,### $p < 0.001$ by Student's t-test; n = 4 independent experiments). (**d**) Mutants display reduced acetylcholine release at the worm neuromuscular junction. Young adult worms expressing WT or mutant UNC-18 were exposed to aldicarb and paralysis was measured every 10 minutes. Data are means ± SEM (n = 6 independent experiments on 20 worms per experiment). (**e, f**) Generation of CRISPR/Cas9-edited P334L (e) and R405H (f) knock-in worms. Successful integration events were screened for by digestion with PstI (e, left panel) or BbsI (f, left panel) and sequencing (e and f, right panels). (**g**) R405H worms display reduced acetylcholine release at the worm neuromuscular junction, while P334L worms have an increased release. Worms were analyzed as in (d). Data are means ± SEM (n = 6 independent experiments on 20-25 worms per experiment).

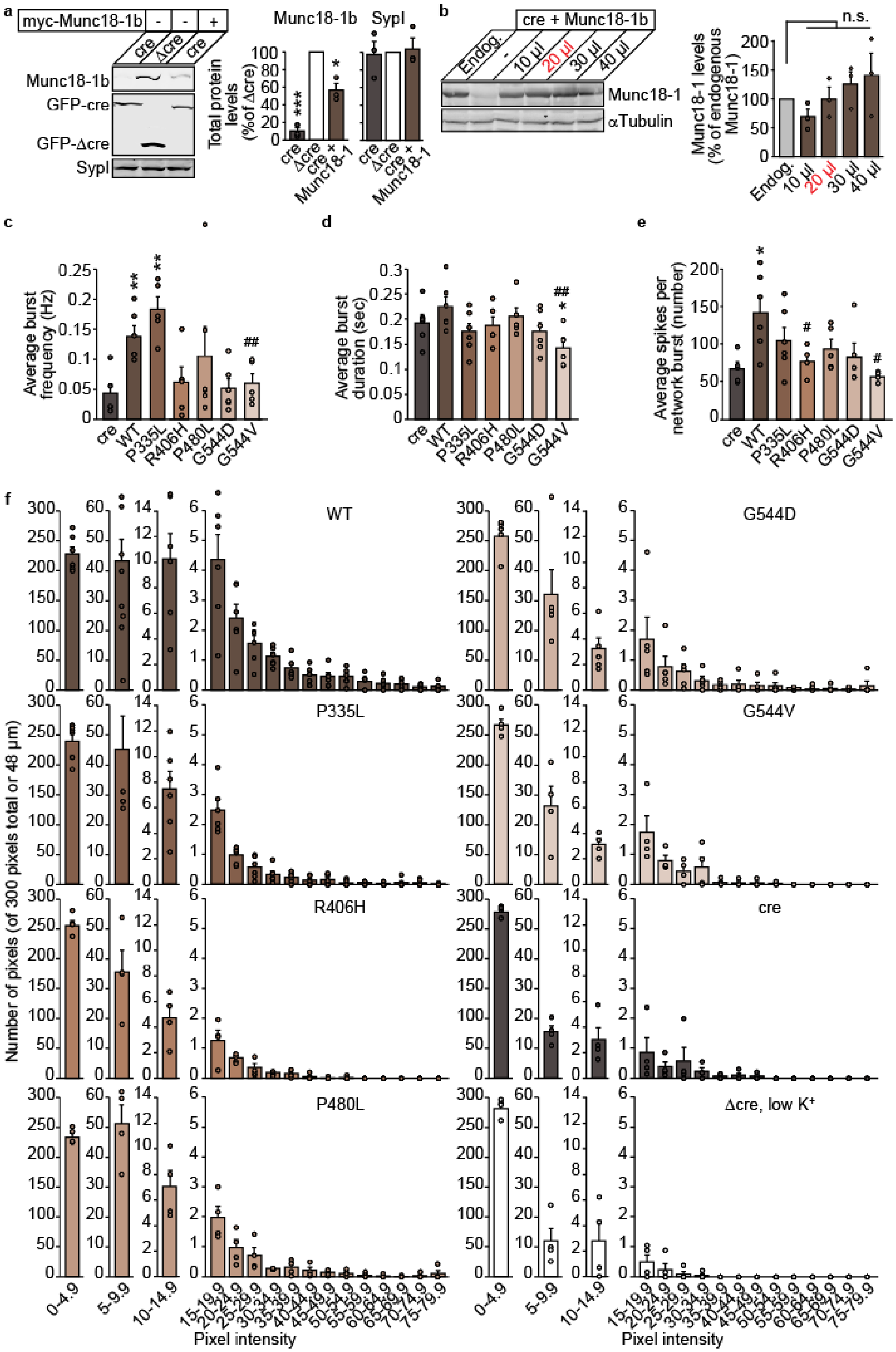


**Supplementary Figure 3. Analysis of synaptic function.** (**a**) Munc18-1 expression levels in Munc18-1 knockout neurons. Primary cortical neurons were infected at 6 days

*in vitro* (DIV) with lentivirus expressing cre recombinase (cre), Δcre (an inactive version of cre; control), or cre with myc-tagged WT Munc18-1. Neurons were harvested at 13 DIV, and total levels of Munc18-1 and synaptophysin (Syp1; a synaptic marker) were analyzed by quantitative immunoblotting. Data are means ± SEM (* $p < 0.05$, *** $p < 0.001$ by Student's t-test; n = 3 independent experiments). (**b**) Lentiviral expression of Munc18-1b leads to endogenous Munc18-1 expression levels. Primary cortical neurons were infected with lentivirus expressing cre and increasing amounts of lentivirus expressing myc-tagged WT Munc18-1 (note: all other experiments use 20 µl of lentivirus). Neurons were harvested at 13 DIV, and total levels of Munc18-1 and α-tubulin were analyzed by quantitative immunoblotting. Data are means ± SEM (n = 3 independent experiments). (**c-e**) Synaptic impairments in primary mouse neurons expressing Munc18-1 variants. Primary neurons were plated on a multielectrode array and were infected with lentiviral vectors expressing cre and WT or mutant Munc18-1. Neurons were subjected to analysis of average burst frequency, average burst duration, and average spikes per network burst. Data are means ± SEM ($^{*,\#}$ $p < 0.05$, $^{**,\#\#}$ $p < 0.01$ by Student's t-test; n = 4-6 independent experiments). (**f**) Distribution of pixel intensities in primary neurons expressing wild-type or mutant Munc18-1 that were subjected to an antibody uptake assay. Primary cortical neurons infected at 6 DIV with lentivirus expressing cre recombinase and/or wild-type or mutant Munc18-1b variants, were subjected to an antibody uptake assay at 13 DIV (Δcre, low $K^+$ = no stimulation control of WT neurons). Endocytosed synaptotagmin-1 antibody was quantified by immunostaining, via counting the number of pixels in pixel intensity bins of 5. Data are means ± SEM (n = 4 independent experiments).

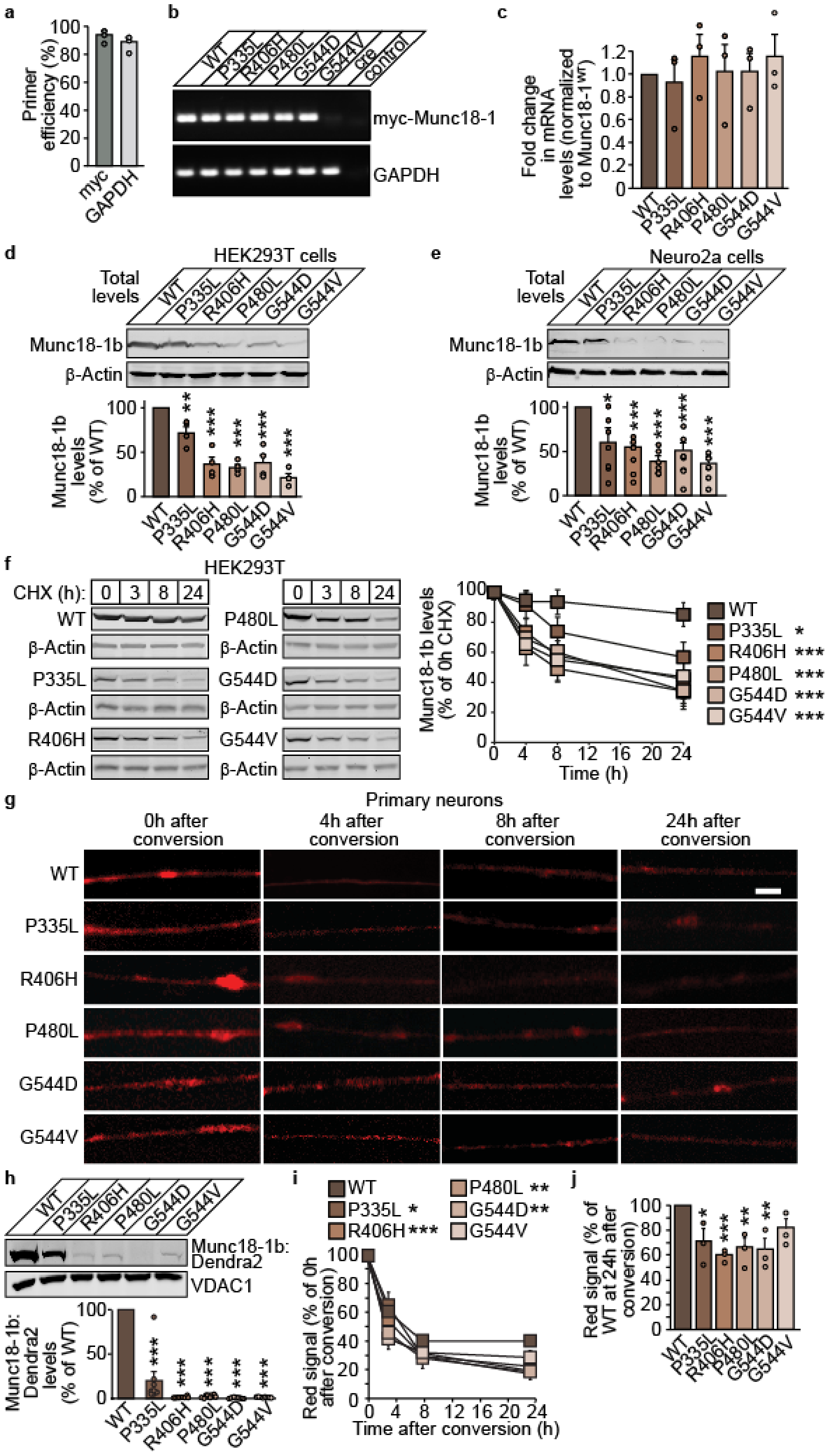


**Supplementary Figure 4. Increased turnover of Munc18-1 mutants in heterologous cells and primary mouse neurons.** (**a-c**) Analysis of Munc18-1 mRNA

levels. Primary cortical neurons were infected with lentiviral vectors expressing cre and WT or mutant Munc18-1 at 6 DIV. Neurons were harvested at 13 DIV and subjected to RNA isolation and qPCR analysis using gel electrophoresis (b) and the ΔΔCt method (c) at same primer efficiency (a). Data are means ± SEM (n = 3 independent experiments). (**d, e**) Total proteins levels of Munc18-1b. Wild-type (WT) or mutant Munc18-1 were expressed in HEK293T cells (d) or Neuro2a cells (e). Two days after transfection, cells were harvested and analyzed for total protein levels, normalized to β-actin. Data are means ± SEM (* $p < 0.05$, ** $p < 0.01$, *** $p < 0.001$ by Student's t-test; n = 4-7 independent experiments). (**f**) Turnover of Munc18-1b. HEK293T cells expressing Munc18-1 WT or mutants were subjected to a cycloheximide (CHX) chase experiment for the indicated time, to stop protein translation. Remaining protein levels were quantified by immunoblotting, normalized to β-actin levels. Data are means ± SEM (* $p < 0.05$, *** $p < 0.001$ by two-way ANOVA; n = 3 independent experiments). (**g-j**) Turnover of Munc18-1 by Dendra2 photoconversion. Primary cortical neurons were infected with lentiviral vectors expressing cre and WT or mutant Munc18-1b:Dendra2 fusion proteins (h). Seven days after infection, expressed Dendra2 was photoconverted, and the red signal was quantified at 0h, 3h, 8h, and 24h after photoconversion (g, i, j). Data are means ± SEM (* $p < 0.05$, ** $p < 0.01$, *** $p < 0.001$ by Student's t-test in (h) and (**j**), and by two-way ANOVA in (i); n = 3-8 independent experiments). Scale bar in (g) = 50 µm.

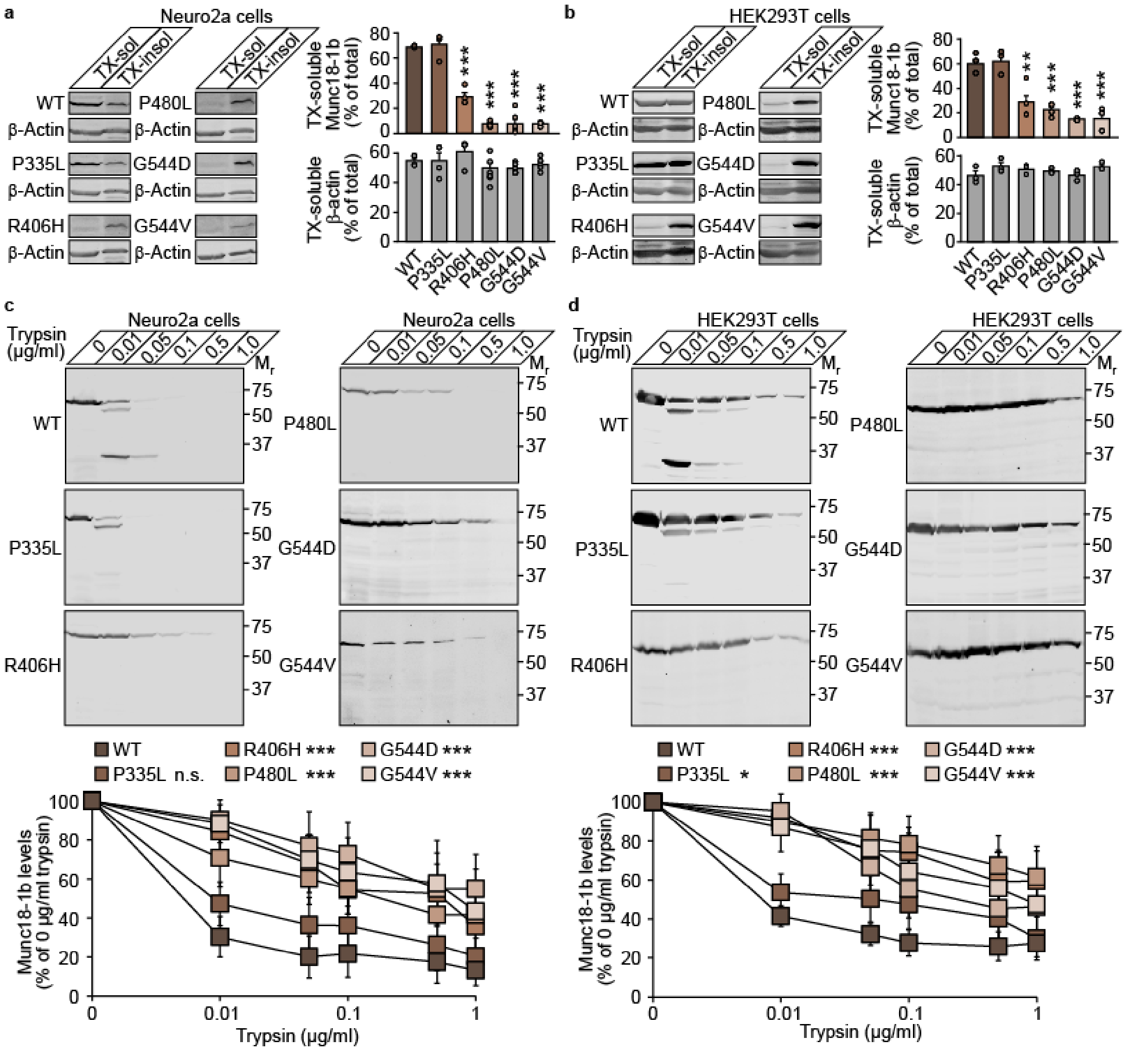


**Supplementary Figure 5. Munc18-1 mutants are less soluble than wild-type Munc18-1.** (**a, b**) Solubility of Munc18-1. Wild-type (WT) and mutant Munc18-1 were expressed in Neuro2a cells (a) or HEK293T cells (b). Two days after transfection, cells were solubilized in 0.1% Triton X-100 (TX). Equal volumes of soluble and insoluble fractions were separated by SDS-PAGE, and TX-soluble Munc18-1 and β-actin (control) was measured as percent of total Munc18-1 by quantitative immunoblotting. Data are means ± SEM (** $p < 0.01$, *** $p < 0.001$ by Student's t-test; n = 3 independent experiments). (**c, d**) Limited proteolysis of Munc18-1. WT and mutant Munc18-1 were expressed in Neuro2a cells (c) or HEK293T cells (d). Two days after transfection, cells were subjected to proteolysis using the indicated concentrations of trypsin. Remaining protein levels were quantified by immunoblotting. Data are means ± SEM (* $p < 0.05$, *** $p < 0.001$ by two-way ANOVA; n = 3 independent experiments; n.s. = not significant).

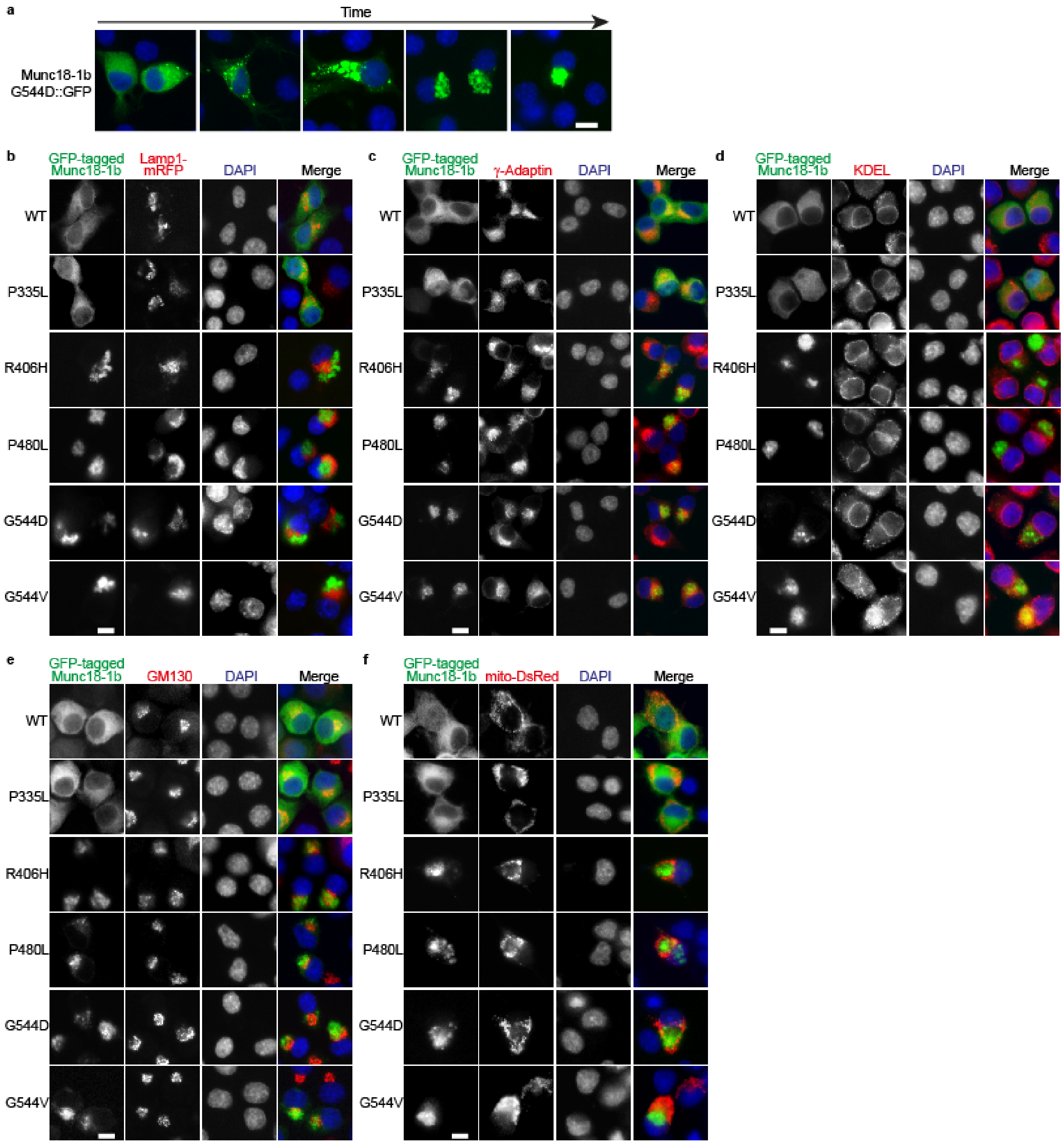


**Supplementary Figure 6. Munc18-1 aggregates grow over time and do not co-localize with organellar markers.** (**a**) Aggregates of Munc18-1 increase in size over time. Neuro2a cells were transfected with G544D Munc18-1, and cells were fixed at 10h, 14h, 18h, 22h, and 38h post-transfection (green = Munc18-1; blue = nuclear marker DAPI). (**b-f**) Subcellular localization of Munc18-1 aggregates. Wild-type (WT) or mutant Munc18-1 were expressed in Neuro2a cells. Two days after transfection, cells were fixed and Munc18-1 localization was visualized as GFP fluorescence. Co-localization with lysosomes (Lamp1-RFP), the trans-Golgi network (γ-Adaptin), the endoplasmic reticulum (KDEL), the cis Golgi network (GM130), mitochondria (mito-DsRed) or nuclei (DAPI). Scale bar = 10 μm.

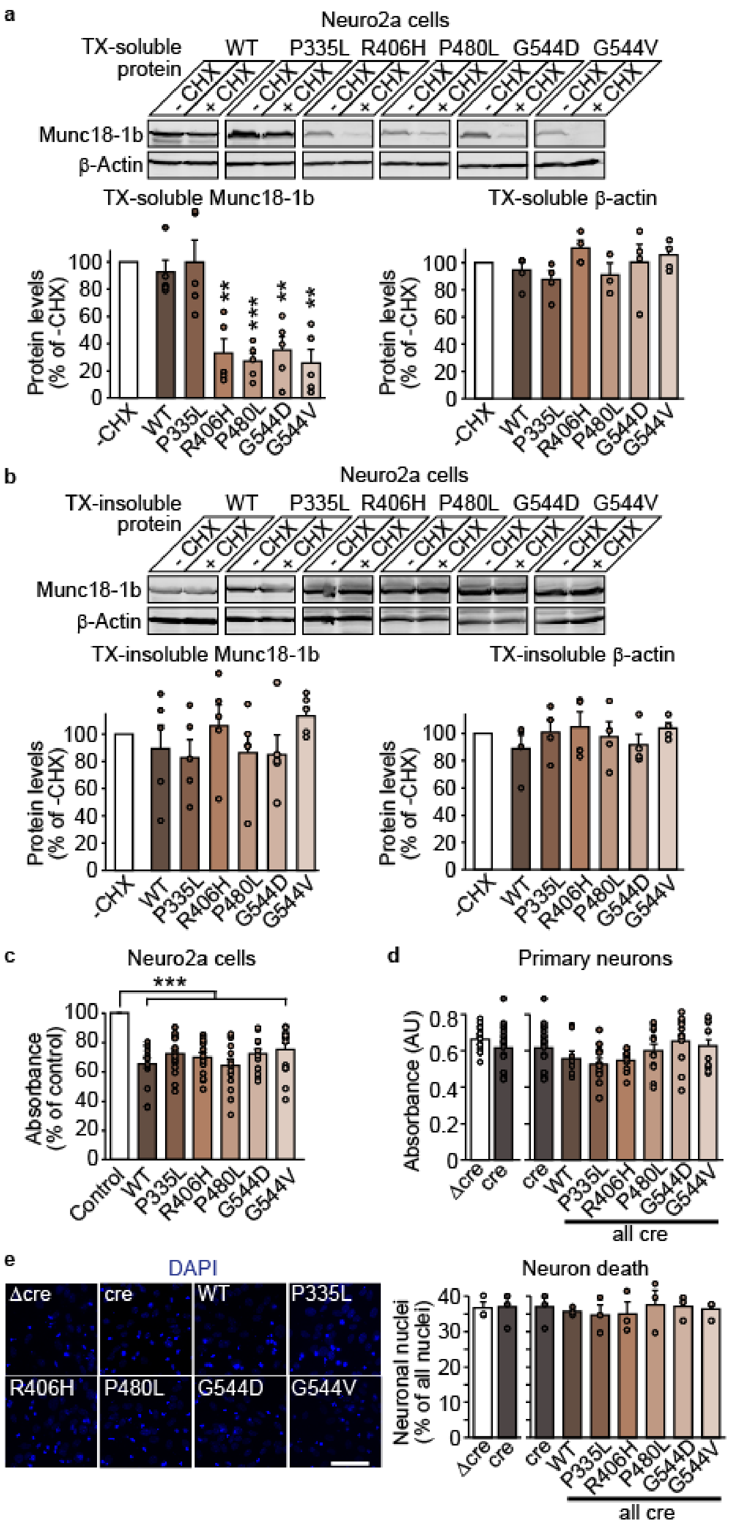


**Supplementary Figure 7. Munc18-1-containing aggregates are stable and not toxic to cells.** (**a, b**) Turnover of soluble and insoluble Munc18-1. Wild-type (WT) or mutant Munc18-1 were expressed in Neuro2a cells. Two days after transfection, cells

were subjected to a cycloheximide (CHX) chase experiment for 24h. Triton X-100 soluble (a) and insoluble (b) protein was measured before and after addition of CHX. Data are means ± SEM (** $p < 0.01$, *** $p < 0.001$ by Student's t-test; n = 5 independent experiments). (**c**) Metabolic activity of Neuro2a cells transfected with WT or mutant Munc18-1. Two days after transfection, cells were subjected to an MTT assay. Data were normalized to untransfected cells, and are means ± SEM (*** $p < 0.001$ by Student's t-test; n = 15 independent experiments). (**d**) Metabolic activity of primary cortical neurons expressing WT or mutant Munc18-1. Seven days after infection, neurons were subjected to an MTT assay. Neurons expressing cre or Δcre served as control. Data are means ± SEM (n = 8-12 independent experiments). (**e**) Neuronal survival of primary cortical neurons expressing WT or mutant Munc18.1. Seven days after infection, neurons were fixed and DAPI-positive neuronal nuclei were counted as percent of all nuclei. Neurons expressing cre or Δcre served as control. Data are means ± SEM (n = 3 independent experiments). Scale bar = 60 µm.

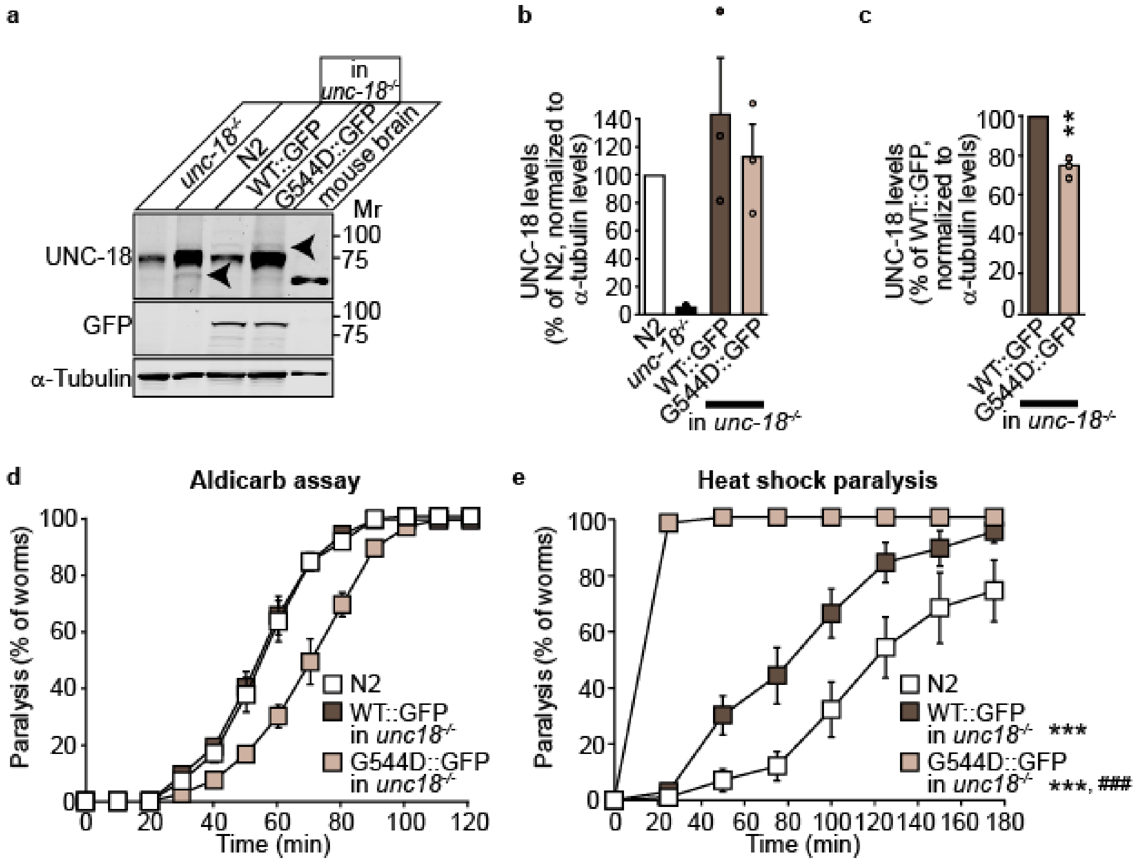


**Supplementary Figure 8. UNC-18 levels in *C. elegans* expressing GFP-tagged WT and G544D.** (**a-c**) Total levels of endogenous UNC-18 (b; lower arrow in panel a) or GFP-tagged UNC-18 (c; higher arrow in panel a) were analyzed by quantitative immunoblotting, normalized to α-tubulin levels. Mouse brain homogenate was separated at the same time, because the Munc18-1 antibody bound non-specifically to other proteins in worm homogenates. Data are means ± SEM (** $p < 0.01$ by Student's t-test; n = 3 independent experiments). (**d**) Mutants display reduced acetylcholine release at the worm neuromuscular junction. Young adult worms expressing WT or mutant *unc-18* were exposed to 1 mM aldicarb and paralysis was measured every 10 minutes. Data are means ± SEM (n = 10 independent experiments on 20 worms per experiment). (**e**) Heat-induced paralysis. Indicated worm strains were exposed to 37°C over a period of 180 min, and paralysis was scored at indicated time points. Data are means ± SEM (*** $p < 0.001$ by two-way ANOVA, compared to N2; $^{###}$ $p < 0.001$ by two-way ANOVA, compared to *unc-18* WT::GFP in *unc-18*$^{-/-}$, n = 10 independent experiments on 10 worms per experiment).

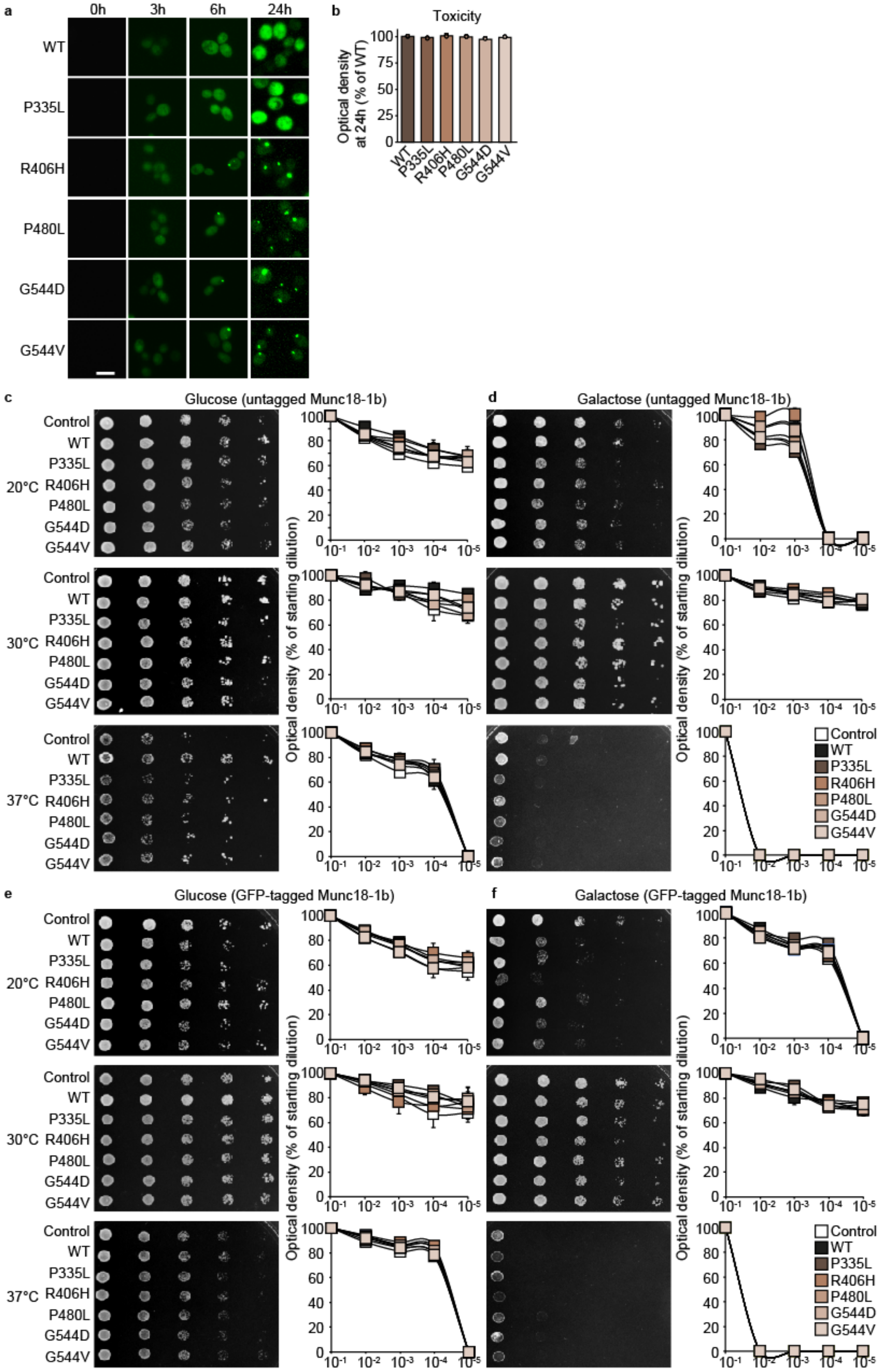


**Supplementary Figure 9. Aggregation and lack of toxicity of mutant Munc18-1 in *S. cerevisiae*.** (**a**) Aggregation of mutant Munc18-1 in yeast. *S. cerevisiae* were

transformed with plasmids expressing GFP-tagged WT or mutant Munc18-1. Yeast cells were imaged at indicated time points after induction of protein expression with galactose. Scale bar = 5 µm. (**b-f**) Lack of toxicity of mutant Munc18-1 in yeast. Proteins were expressed as described above, and cell density was measured at 24h post-induction of protein expression (b). Alternatively, yeast transformed with WT or mutant expression vectors were spotted at ten-fold serial dilutions onto ura- plates containing glucose (c, e; control) or galactose (d, f; induction of protein expression). Plates were imaged after 2 days at 20°C, 30°C, or 37°C, and optical density was analyzed as a function of the starting dilution. Data are means ± SEM (n = 4 independent experiments).

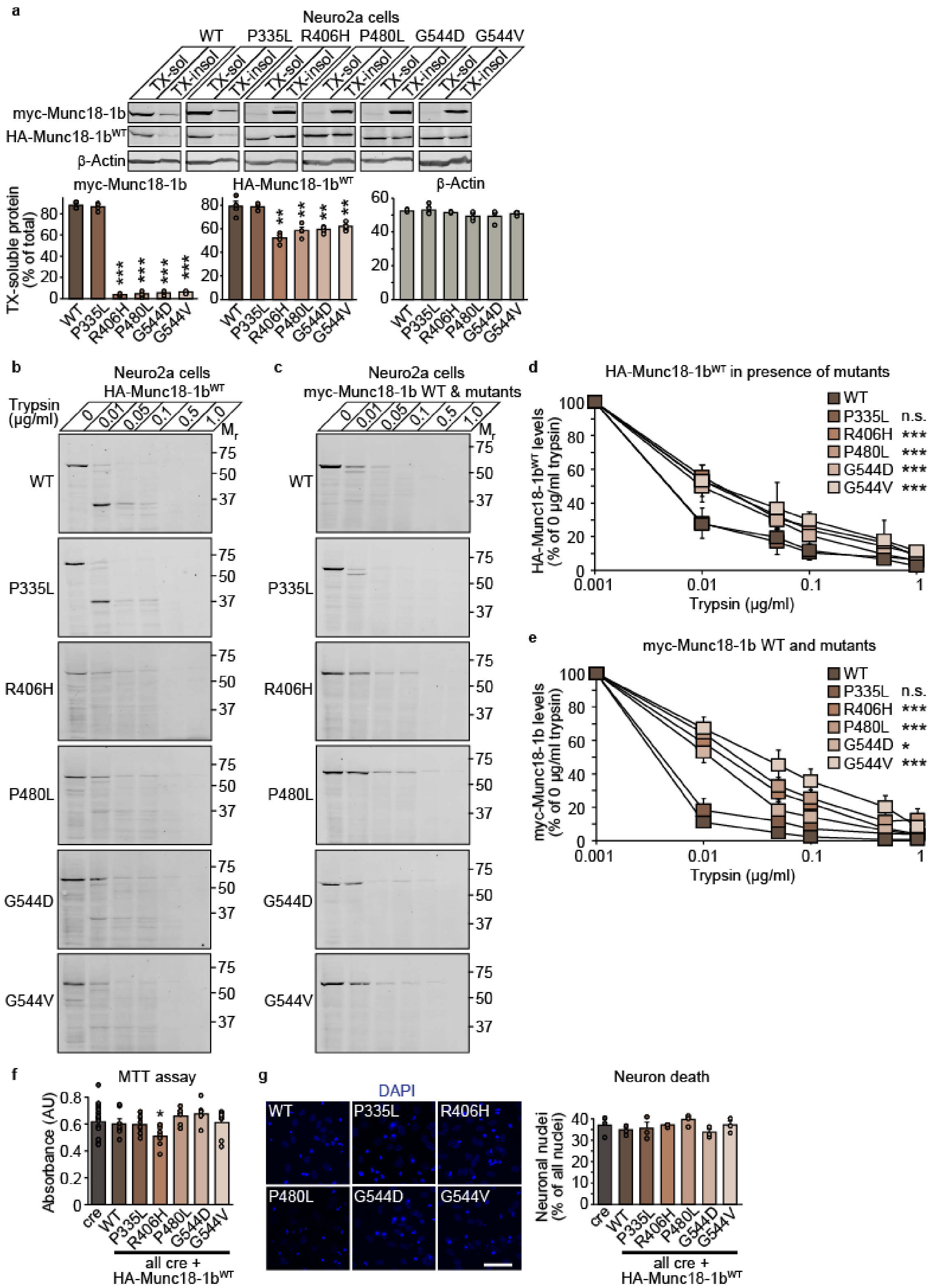


**Supplementary Figure 10. Dominant negative activity of mutant Munc18-1 on wild-type Munc18-1 in Neuro2a cells.** (**a**) Solubility of Munc18-1 in Neuro2a cells. Neuro2a

cells were co-transfected with myc-tagged wild-type (WT) or mutant Munc18-1 and HA-tagged WT Munc18-1. Two days after transfection, cells were solubilized in 0.1% Triton X-100 (TX). Equal volumes of TX-soluble and -insoluble fractions were separated by SDS-PAGE, and TX-soluble myc-tagged Munc18-1, HA-tagged Munc18-1 and β-actin (control) was measured as percent of total protein by quantitative immunoblotting. Data are means ± SEM (** $p < 0.01$, *** $p < 0.001$ by Student's t-test; n = 4 independent experiments). (**b-e**) Limited proteolysis of Munc18-1. Myc-tagged WT or mutant Munc18-1 were co-expressed with HA-tagged WT Munc18-1 in Neuro2a cells. Two days after transfection, cells were subjected to proteolysis using the indicated concentrations of trypsin. Remaining protein levels were quantified by immunoblotting. Data are means ± SEM (* $p < 0.05$, *** $p < 0.001$ by two-way ANOVA; n = 3 independent experiments; n.s. = not significant). (**f**) Metabolic activity of primary cortical neurons expressing WT and mutant Munc18-1. Seven days after infection, neurons infected with lentiviral vectors expressing cre, myc-tagged Munc18-1b WT or mutants, and HA-tagged Munc18-1b WT were subjected to an MTT assay. Data are means ± SEM (* $p < 0.05$ by Student's t-test; n = 8-15 independent experiments). (**g**) Neuronal survival of primary cortical neurons expressing WT or mutant Munc18-1. Seven days after infection, neurons were fixed and DAPI-positive neuronal nuclei were counted as percent of all nuclei. Neurons expressing cre or Δcre served as control. Data are means ± SEM (n = 3 independent experiments). Scale bar = 60 µm.

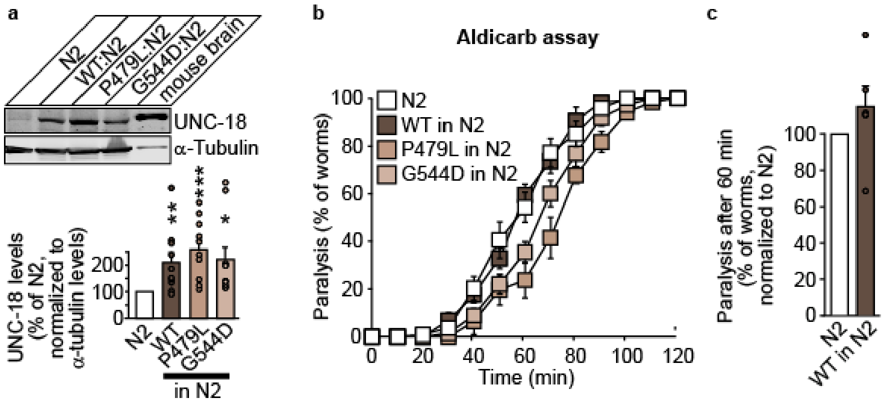


**Supplementary Figure 11. Impairments in neurotransmitter release in *C. elegans* expressing mutant UNC-18 on a wild-type N2 background.** (**a**) Expression levels of UNC-18 in *C. elegans*. Total levels of UNC-18 were analyzed by quantitative immunoblotting, normalized to α-tubulin levels. Mouse brain homogenate was separated at the same time, because the Munc18-1 antibody bound non-specifically to other proteins in worm homogenates. Data are means ± SEM (* $p < 0.05$, ** $p < 0.01$, *** $p < 0.001$ by Student's t-test; n = 10 independent experiments). (**b, c**) Munc18-1 mutants display reduced acetylcholine release at the worm neuromuscular junction. Young adult worms expressing WT or mutant *unc-18* were exposed to aldicarb and paralysis was measured every 10 minutes (b). There is no significant difference in the aldicarb assay between N2 worms and N2 worms expressing WT UNC-18 (c). Data are means ± SEM (n = 6 independent experiments on 20 worms per experiment).

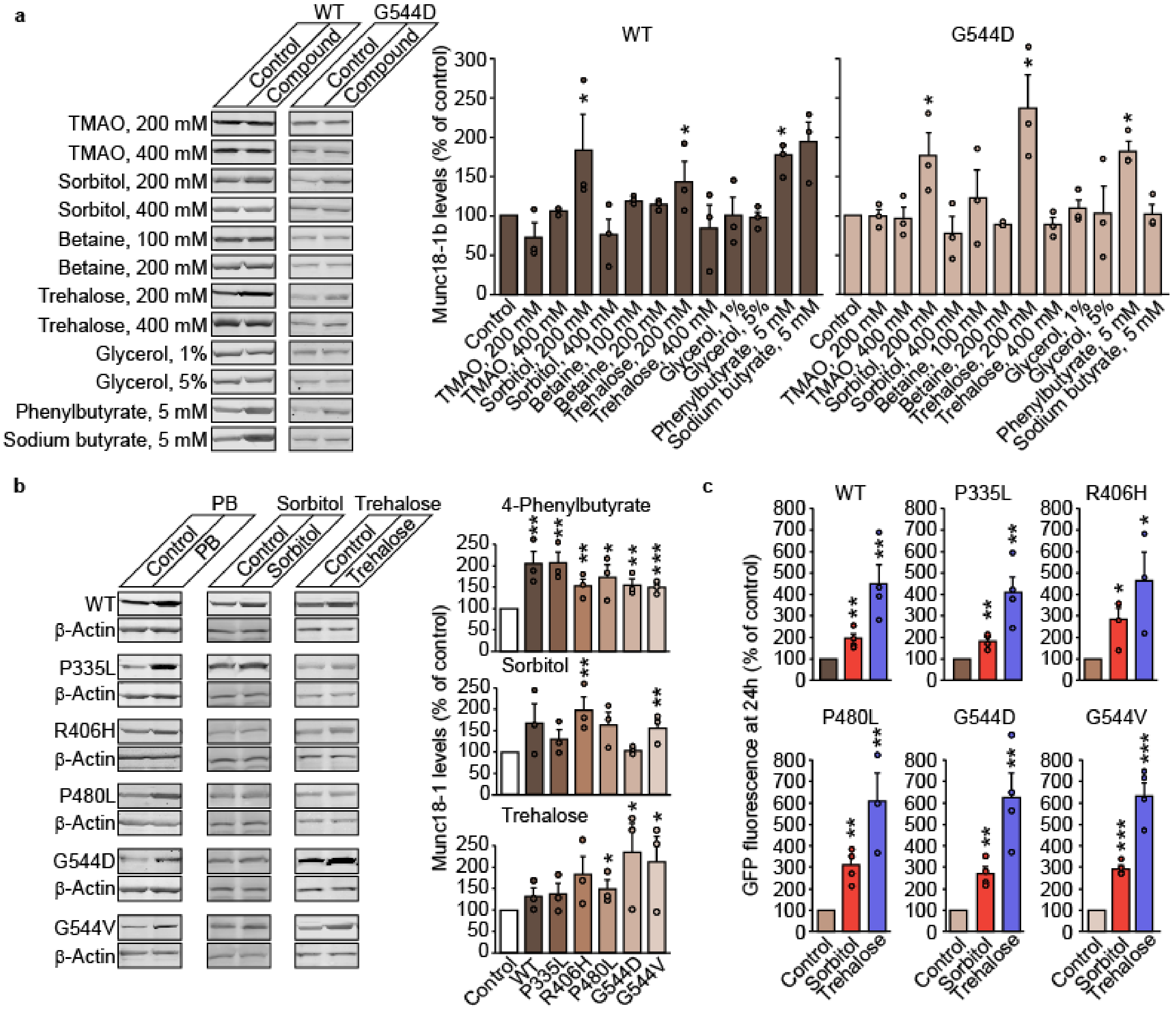


**Supplementary Figure 12. Rescue of Munc18-1 protein levels with chemical chaperones.** (**a**) Rescue of Munc18-1 levels. HEK293T cells were transfected with cDNA expressing WT or G544D Munc18-1 in absence or presence of the indicated chemical chaperone. Total protein levels were quantified 2 days after transfection by quantitative immunoblotting, normalized to the control. Data are means ± SEM (* $p < 0.05$ by Student's t-test; n = 3 independent experiments). (**b**) Same as in (a), except that HEK293T cells were transfected with plasmids expressing WT or mutant Munc18-1 in absence or presence of 5 mM 4-phenylbutyrate, 200 mM sorbitol, or 200 mM trehalose. Data are means ± SEM (* $p < 0.05$, ** $p < 0.01$, *** $p < 0.001$ by Student's t-test; n = 3 independent experiments). (**c**) Munc18-1 levels in yeast. Protein expression was induced with galactose in absence or presence of 400 mM sorbitol or trehalose, and Munc18-1 levels were analyzed by measuring GFP fluorescence in a plate reader 24h after induction. Data are means ± SEM (** $p < 0.01$, *** $p < 0.001$ by Student's t-test; n = 3-4 independent experiments).

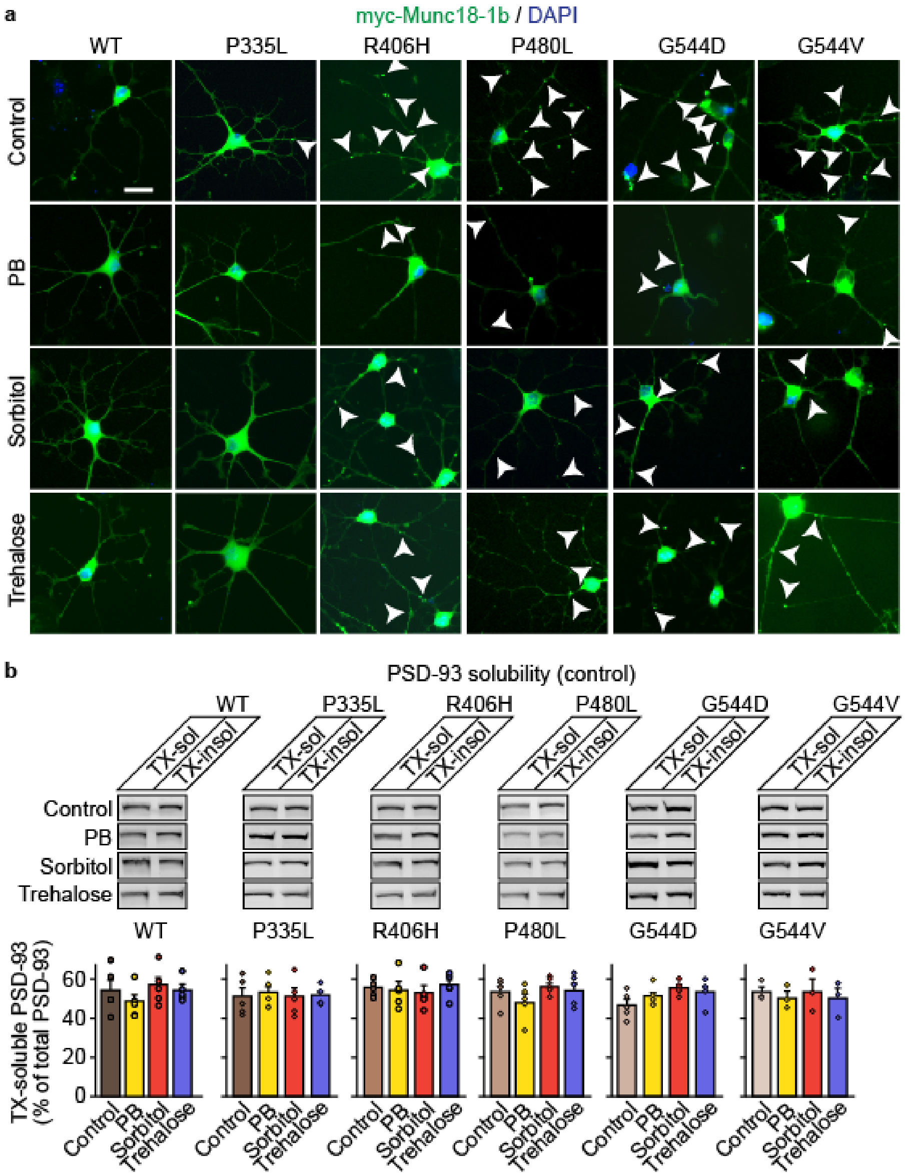


**Supplementary Figure 13. Chemical chaperones rescue mutant Munc18-1 deficits in primary neurons.** (**a**) Aggregation of mutant Munc18-1. Primary Munc18-1 null neurons lentivirally expressing WT or mutant Munc18-1b in absence or presence of chemical chaperones were analyzed for the subcellular localization of Munc18-1b by immunocytochemistry 7 days after infection. Arrows depict aggregates. Scale bar = 20 µm. (**b**) Triton X-100 solubility of PSD-93. WT or mutant Munc18-1b were expressed in primary cortical neurons infected with lentiviral vectors expressing cre recombinase and Munc18-1b variants in presence or absence of chemical chaperones. Seven days after infection, cells were solubilized in 0.1% Triton X-100 (TX). Equal volumes of soluble and insoluble fractions were separated by SDS-PAGE, and TX-soluble PSD-93 was measured as percent of total PSD-93 by quantitative immunoblotting. Data are means ± SEM (n = 5 independent experiments).

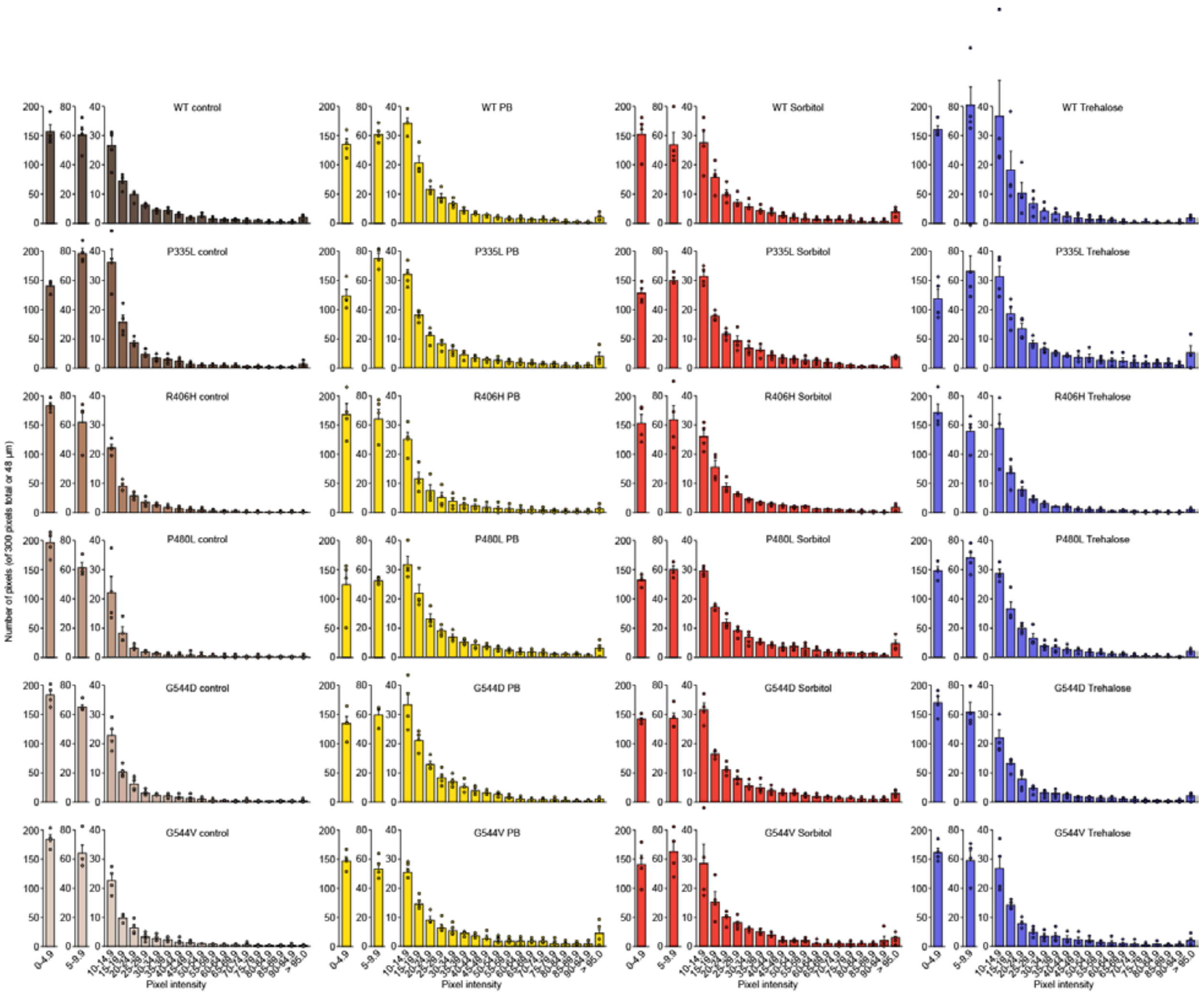


**Supplementary Figure 14. Antibody uptake assay.** Distribution of pixel intensities in primary neurons expressing wild-type (WT) or mutant Munc18-1 that were subjected to an antibody uptake assay in absence or presence of chemical chaperones. Primary cortical neurons infected at 6 days *in vitro* (DIV) with lentivirus expressing cre recombinase and/or WT or mutant Munc18-1b variants, were subjected to an antibody uptake assay at 13 DIV. Endocytosed synaptotagmin-1 antibody was quantified by immunostaining, via counting the number of pixels in pixel intensity bins of 5. Note, that a different batch of synaptotagmin-1 antibody was used for this Figure compared to Figure 2. Data are means ± SEM (n = 4 independent experiments).

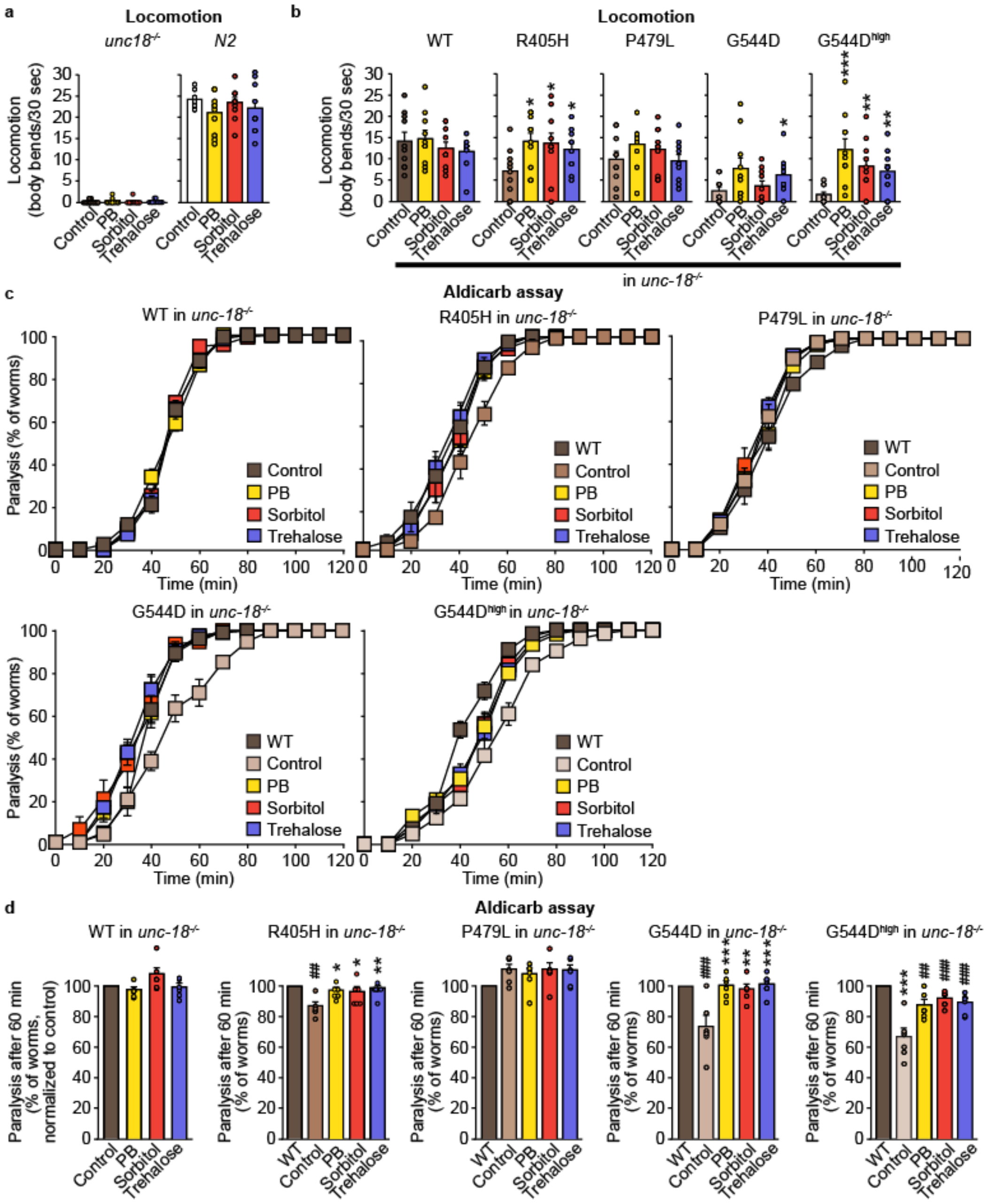


**Supplementary Figure 15. Chemical chaperones rescue deficits of mutant UNC-18 in locomotion and acetylcholine release in *C. elegans*.** (**a, b**) Locomotion of *C. elegans*. Worms maintained for 2-3 generations on plates containing or lacking chemical chaperones were transferred to an agar plate without food source, were poked once with a platinum-iridium wire, and body bends per 30 sec were counted. Data are means ± SEM (* $p < 0.05$, ** $p < 0.01$, *** $p < 0.001$ by Student's t-test; n = 10 independent experiments on 10 worms per experiment). (**c, d**) Rescue of reduced acetylcholine release in worms expressing mutant UNC-18 variants. Young adult worms expressing WT or mutant *unc-18* that were maintained for 2-3 generations on plates

containing or lacking chemical chaperones, were exposed to aldicarb and paralysis was scored every 10 min (c) or measured at 60 min (d). Data are means ± SEM (* $p < 0.05$, $^{**,\#\#}$ $p < 0.01$, $^{***,\#\#\#}$ $p < 0.001$ by Student's t-test in (d); n = 6 independent experiments on 20 worms per experiment).

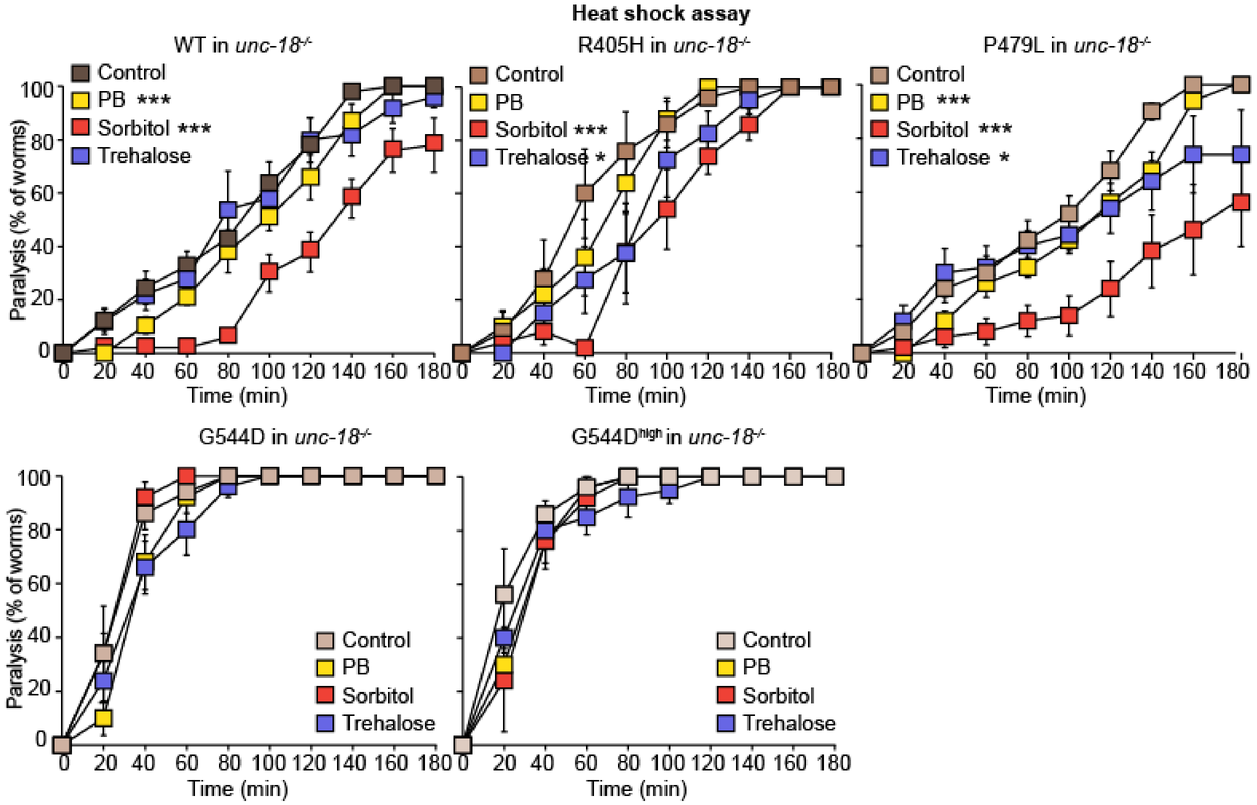


**Supplementary Figure 16. Chemical chaperones delay paralysis during heat shock of mutant UNC-18 in *C. elegans*.** Rescue of heat-induced paralysis. Indicated worm strains that were maintained for 2-3 generations on plates containing or lacking chemical chaperones were exposed to 37°C over a period of 180 min, and paralysis was scored at indicated time points. Data are means ± SEM (* $p < 0.05$, *** $p < 0.001$ by two-way ANOVA, compared to control, n = 5-6 independent experiments on 10 worms per experiment).

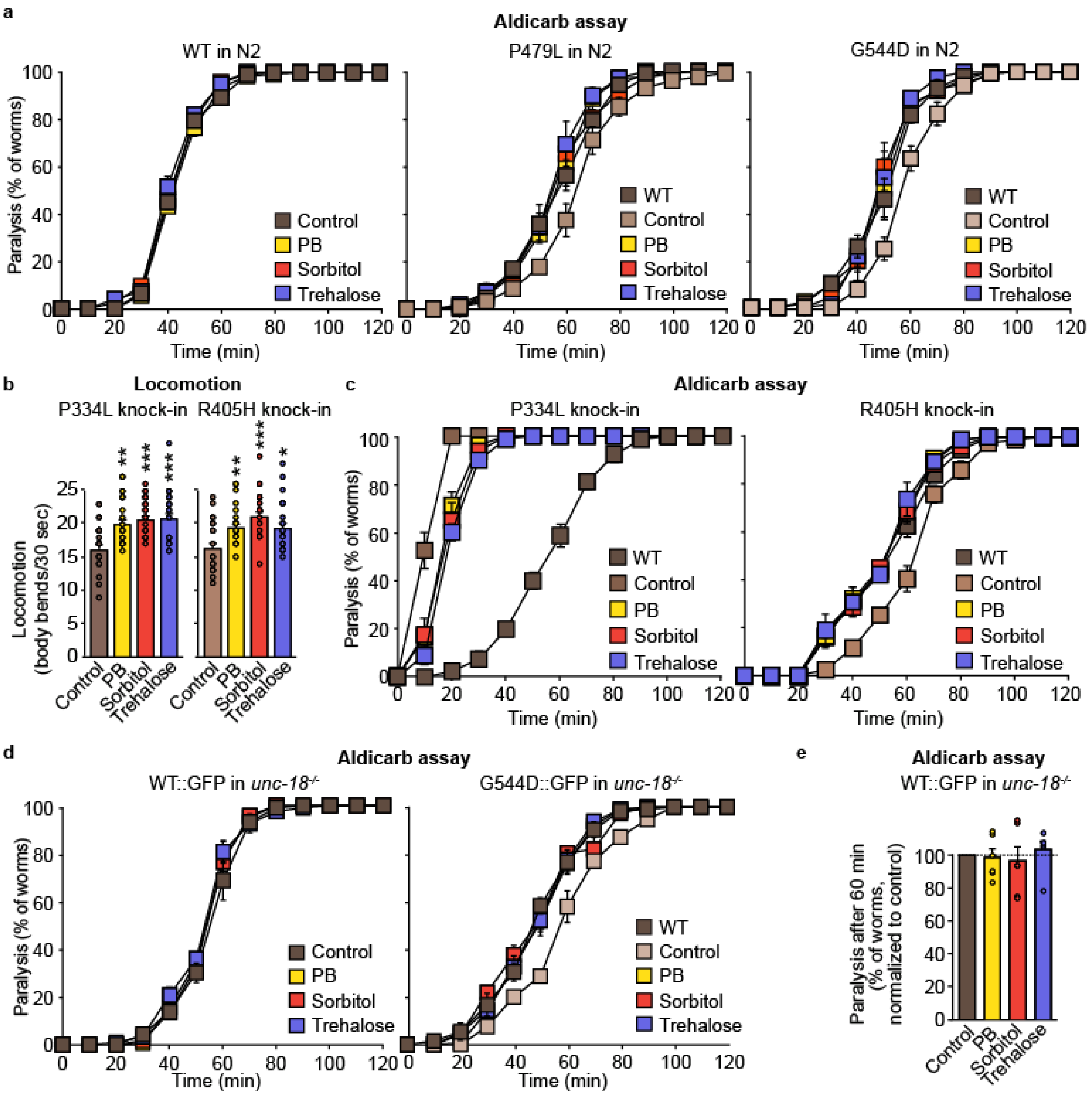


**Supplementary Figure 17. Chemical chaperones rescue deficits of mutant UNC-18 in acetylcholine release in *C. elegans*.** (**a**) Rescue of reduced acetylcholine release in worms expressing mutant UNC-18 variants. Young adult worms expressing WT or mutant *unc-18* that were maintained for 2-3 generations on plates containing or lacking chemical chaperones, were exposed to aldicarb and paralysis was scored every 10 min. Data are means ± SEM (n = 6 independent experiments on 20 worms per experiment). (**b**) Locomotion of *C. elegans*. Worms maintained for 2-3 generations on plates containing or lacking chemical chaperones were transferred to an agar plate without food source, were poked once with a platinum-iridium wire, and body bends per 30 sec were counted. Data are means ± SEM (* $p < 0.05$, ** $p < 0.01$, *** $p < 0.001$ by

Student’s t-test; n = 20 worms). (**c**) Same as (a), except that CRISPR/Cas9-generated knock-in worms were used. Data are means ± SEM (n = 4 independent experiments on 20 worms per experiment). (**d, e**) Same as (a), except that worms expression GFP-tagged wild-type and mutant UNC-18 variants were analyzed. Data are means ± SEM (n = 6 independent experiments on 20 worms per experiment).

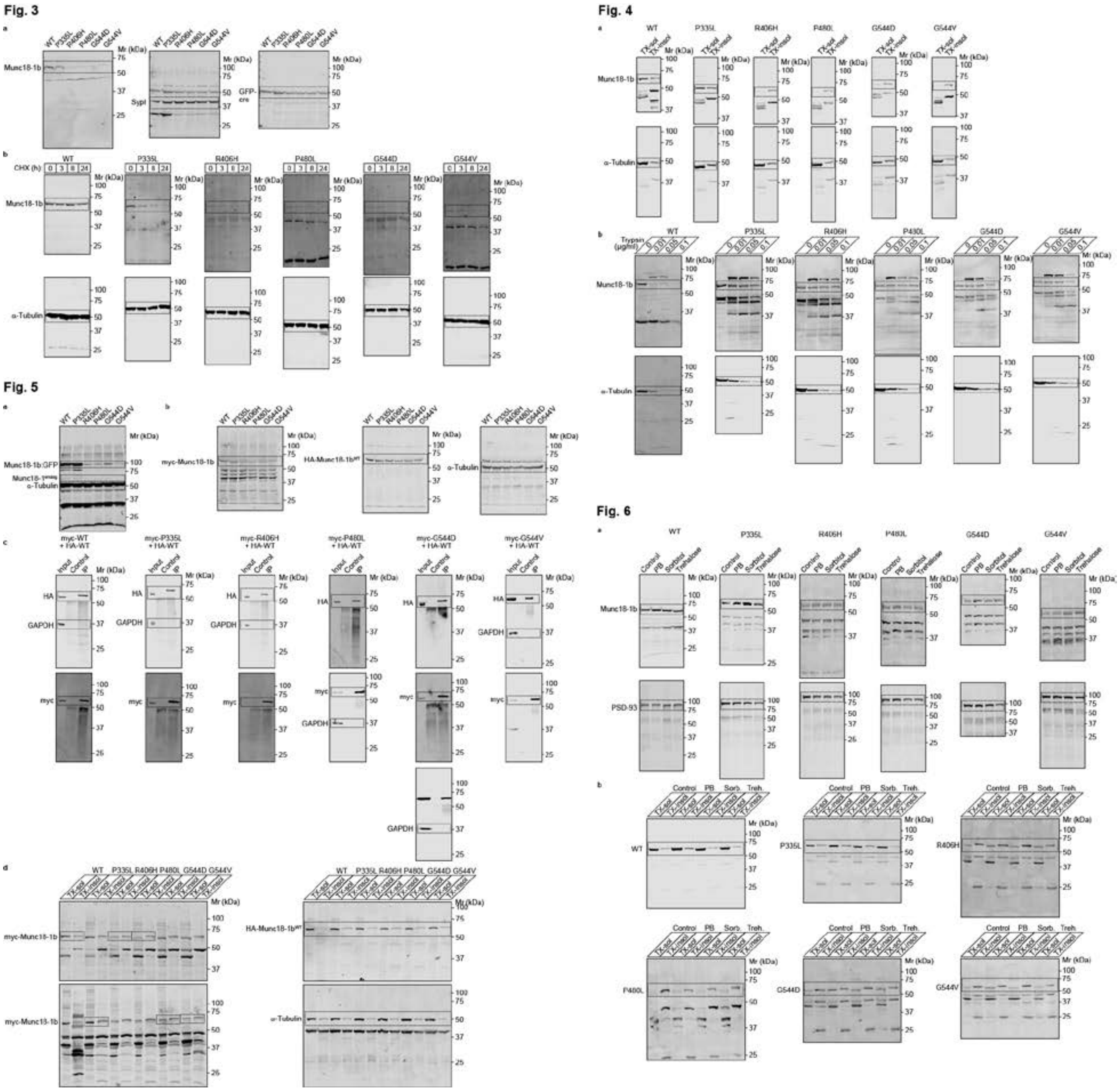


**Supplementary Figure 18.** Full-length pictures of the blots presented in the main figures.

**Supplementary References**

1. Colbert, K.N. *et al*. Syntaxin1a variants lacking an N-peptide or bearing the LE mutation bind to Munc18a in a closed conformation. *Proc Natl Acad Sci U S A* **110**, 12637-12642 (2013).
